# Perpetration of intimate partner violence and mental health outcomes: sex- and gender-disaggregated associations among adolescents and young adults in Nigeria

**DOI:** 10.7189/jogh.10.010708

**Published:** 2020-06

**Authors:** Lindsay Stark, Ilana Seff, Ann M Weber, Beniamino Cislaghi, Melissa Meinhart, Laura Gauer Bermudez, Victor Atuchukwu, Dennis Onotu, Gary L Darmstadt

**Affiliations:** 1 George Warren Brown School, Washington University in St. Louis, St. Louis, Missouri, USA; 2 Department of Population and Family Health, Columbia University Mailman School of Public Health, New York, New York, USA; 3 Department of Pediatrics, Stanford University School of Medicine, Stanford, CA, USA; 4 London School of Hygiene and Tropical Medicine, London, UK; 5 Columbia University School of Social Work, New York, New York, USA; 6 US Centers for Disease Control and Prevention, Abuja, Nigeria

## Abstract

**Background:**

The association between intimate partner violence (IPV) victimisation and poor mental health outcomes is well established. Less is known about the correlation between IPV perpetration and mental health, particularly among adolescents and young adults. Using data from the nationally representative Violence Against Children Survey, this analysis examines the association between IPV perpetration and mental health for male and female adolescents and young adults in Nigeria.

**Methods:**

Multivariate logistic regression models were used to examine associations between ever-perpetration of IPV and four self-reported mental health variables: severe sadness, feelings of worthlessness, suicide ideation, and alcohol use. Models were sex-disaggregated, controlled for age, marital status, and schooling, and tested with and without past exposure to violence. Standard errors were adjusted for sampling stratification and clustering. Observations were weighted to be representative of 13-24 year-olds in Nigeria.

**Results:**

Males were nearly twice as likely as females to perpetrate IPV (9% v. 5%, respectively; *P* < 0.001), while odds of perpetration for both sexes were higher for those ever experiencing IPV (adjusted odds ratio (aOR) = 4.60 for males; aOR = 2.71 for females). Female perpetrators had 2.73 higher odds of reporting severe sadness (95% confidence interval CI = 1.44, 5.17; *P* = 0.002) and 2.72 times greater odds of reporting suicide ideation (1.28, 5.79; *P* = 0.010) than non-perpetrating females, even when controlling for past-year violence victimisation. In contrast, male perpetrators had 2.65 times greater odds of feeling worthless (1.09, 6.43; *P* = 0.031), and 2.36 times greater odds of reporting alcohol use in the last 30 days (1.50, 3.73; *P* < 0.001), as compared to non-perpetrating males.

**Conclusions:**

Among adolescents and young adults in Nigeria, IPV perpetration and negative mental health outcomes are associated but differ for males and females. Mindful of the cross-sectional nature of the data, it is possible that socially determined gender norms may shape the ways in which distress from IPV perpetration is understood and expressed. Additional research is needed to clarify these associations and inform violence prevention efforts.

While the association between violence victimisation and poor mental health outcomes has been well established [1,2], less is known about the correlation between violence perpetration and mental health outcomes, particularly among male and female adolescents and young adults in low- and middle-income countries (LMICs) [3]. To date, the social, economic, and health correlates of intimate partner violence (IPV) perpetration have been most markedly researched from the perspective of adult male perpetrators [4,5]. These studies demonstrate that adult males’ perpetration of IPV is correlated with substance use, adverse mental health, and previous exposure to violence.

Within the smaller body of literature that investigates female IPV perpetration, studies have tended to focus on mutually violent relationships [6,7] or lack sex-disaggregated associations between perpetration and mental health [8]. The few studies that include associations for female IPV perpetrators come from high-income countries and have indicated a positive correlation between the perpetration of IPV and depressive symptoms for both males and females [3,9]. The mental health impacts of violence perpetration have been shown to be more severe among females than males [9].

The limited LMIC studies on gender-based violence (GBV) more broadly (perpetrated by both intimate partners and non-partners), generally align with findings from upper income countries, demonstrating high rates of adverse mental health among male perpetrators [10-13]. However, all of these studies focus on male perpetrators, few use population-based sampling methods, none are nationally representative, and none specifically focus on IPV. In addition, these studies fail to account for the full range of violence exposure status (victimisation vs perpetration vs both) and the gendered associations between perpetration and mental health.

While there is an ever-increasing body of literature regarding the impacts of IPV on survivors, empirical research regarding perpetration is scant and even more limited when exploring IPV in sub-Saharan Africa or among female perpetrators. To address these limitations, this paper presents data from the Violence Against Children Survey (VACS), examining the relationship between IPV perpetration and mental health outcomes among a nationally representative sample of male and female adolescents and young adults, ages 13-24 years, in Nigeria. By exploring the association with four mental health variables – severe sadness, feelings of worthlessness, suicide ideation, and alcohol use – for males and females, separately, this paper explores the gendered mental health correlates of IPV perpetration. To our knowledge, this is the first paper to explore the associations between IPV perpetration and mental health for male and female adolescents and young adults in a LMIC setting.

## METHODS

### Setting

Nigeria, the most populous country in Africa, is home to a diverse population that includes more than 250 ethnic groups. While it boasts the largest economy in Africa, social and economic inequities are rife, and it is estimated that nearly half of Nigerians (46%) live below the international poverty line [14]. Gender inequities, due in part to Nigeria’s patriarchal norms, are most evident in educational attainment [15], child marriage [16], and female genital mutilation/cutting (FGM/C) [17]. Further, every six in 10 children experience some form of violence before the age of 18 [18]. Nigeria’s precarious situation is particularly marked in the northeast of the country, as the ongoing seven years of conflict continues to exhaust communities of social and economic resources.

Within this broader context, the mental health burden is high. An estimated 20%-30% of Nigerians are estimated to have mental health concerns [19], yet there is a considerable lack of mental health awareness and resources available within the country [20]. Even less is known about the prevalence of mental health needs among adolescents and young adults in Nigeria, though one study suggests that 20% of 10-17 year-old school-goers have considered suicide [21]. Among people with serious mental disorders in Nigeria, there is an 80% treatment gap [22]. Overall responses to mental health needs in Nigeria have improved in the past decade, due to initiatives such as WHO’s Mental Health Gap Action Programme and Nigeria’s revised Mental Health Policy, but more learning is needed to support the sizable mental health burden and the effectiveness of current initiatives.

### Data

The data for this analysis were collected in 2014 as part of a collaboration between the US Centers for Disease Control and Prevention (CDC), UNICEF, the Nigerian government, and other local, bilateral, and multilateral partners. The VACS, which have been implemented in nine countries as of 2018, provide information on exposure to physical, sexual, and emotional violence among 13-24 year-old males and females. Additionally, questionnaires collect data on demographics, HIV-related knowledge and attitudes, sexual behaviour, and potential determinants and consequences of violence, among other topics [18]. The VACS in Nigeria is the first study from West Africa to collect nationally representative data on sexual, physical, and emotional violence for both male and female adolescents and young adults [23].

The Nigeria VACS was a cross-sectional, nationally representative survey, and employed a multi-stage sampling approach. In the first stage, 353 enumeration areas (EAs) from the 2006 census were randomly selected; in the second stage, 20 respondents were randomly sampled within each EA. The survey used a “split sample” approach, whereby 59% of the EAs were male-only and 41% were female-only. This approach was employed to protect the safety and confidentiality of respondents. For example, this method helped to mitigate the likelihood that a male perpetrator and female survivor of the same incident of IPV were both interviewed. Additionally, the number of male EAs was increased to account for higher levels of expected unavailability and non-response among males as compared to females of this age group. Caregiver consent and informed assent were acquired for sample participants 18 years and under. Consent for respondents 18 and over was obtained directly. Surveys were administered electronically using Electronic Data Capture (EDC) in CSPro and, to the extent possible, all surveys were implemented in a private space. Methodology for the Nigeria VACS was reviewed by the CDC’s Institutional Review Board (IRB) and the in-country National Health Ethics Research Committee at the National Ministry of Health [18].

### Variables of interest

This study assessed the relationship between perpetration of IPV and mental health outcomes for male and female adolescents and young adults, while also controlling for exposure to violence. **Table 1** summarises how IPV perpetration and forms of violence exposure were operationalised. Demographic variables of interest included age, whether an individual was married or living with a partner as if married, and whether the respondent had ever attended school.

**Table 1:** Measures of violence-related correlates of mental health outcomes

Ever perpetration of intimate partner violence:
Reported having ever done one or more of the following to a current or previous boyfriend/girlfriend, romantic partner/husband/wife:
-Punched, kicked, whipped, or beat them?
-Choked, suffocated, tried to drown, or intentionally burn them?
-Forced him/her to have sex when they did not want to
**Physical violence victimisation, last 12 months:**
-Reported having ever been punched, kicked, whipped, beat, choked, suffocated, drowned, or intentionally burned by anyone;
-AND most recent or first incident occurred within last 12 months
**Emotional violence by adult caregiver victimisation, last 12 months:**
-Reported that a parent or adult caregiver has ever “told you that you were not loved, or did not deserve to be loved”, “said they wished you had never been born or were dead”, or “ridiculed you or put you down, for example said that you were stupid or useless”
-AND most recent or first incident occurred within last 12 months
**Sexual violence victimisation, last 12 months:**
-Reported that anyone had ever performed unwanted sexual touching, attempted forced sex, coerced sex, or completed forced sex;
-AND most recent or first incident occurred within last 12 months
**Intimate partner violence victimisation, last 12 months:**
-Reported that a former or current partner had ever punched, kicked, whipped, beat, choked, suffocated, tried to drown, intentionally burned, performed unwanted sexual touching, attempted to force respondent to have sex, coerced respondent into having sex, or forced respondent to have sex;
-AND most recent or first incident occurred within last 12 months

Our analysis assessed four outcomes of interest related to mental health, each of which was operationalised using one VACS survey question. Two outcomes capture symptoms of depression: severe sadness and feelings of worthlessness. Respondents were considered to exhibit feelings of severe sadness if they answered “all the time” or “most of the time” to the question: *During the past 30 days, how often did you feel so sad that nothing could cheer you up?* Respondents were considered to exhibit feelings of worthlessness if they selected one of the same two responses for the question: *During the past 30 days, how often did you feel worthless?* Respondents who selected “some of the time”, “a little of the time” or “none of the time”, for either of these questions were considered defined as not exhibiting the respective symptom. Suicide ideation was operationalised using the question: *Have you ever thought about killing yourself?* Lastly, we constructed a measure of alcohol use from the survey question, *In the past 30 days, on how many days did you drink alcohol to the point that you became drunk?* This measure can take a value from 0 to 30.

### Statistical analysis

Analysis was implemented on a final sample of 2437 males and 1766 females, regardless of partnership status. Information was missing in less than 2% of observations for all variables of interest. We first estimated descriptive statistics for all correlates and outcomes of interest for males and females, separately; differences between genders were identified using adjusted Wald tests.

We then used logistic regressions to estimate the relationships between our correlates of interest and each of the three mental health outcomes; Poisson regressions were used to estimate the relationship between our correlates of interest and our measure of alcohol use. Model 1 estimated the relationship between ever-perpetration of IPV and the corresponding mental health outcome, while controlling for age, marital status, and having ever been to school. Model 2 included the same covariates from Model 1, as well as four covariates representing past-year victimisation, ie, exposure to the four types of violence outlined in **Table 1**. Given the literature supporting the salience of mutual violence or reciprocal IPV (ie, when an individual perpetrates IPV in response to victimisation from his or her partner) we conducted sensitivity analyses to ensure that significant associations between perpetration and outcomes of interest were driven by IPV perpetration, and not by IPV exposure which then resulted in perpetration. Specifically, our sensitivity analyses included estimating the same models described above, but on a subsample of those individuals who reported never having been exposed to IPV, including 2263 males and 1576 females.

All regressions were estimated separately for males and females in order to understand how these relationships may be gendered. Observations were weighted to be representative of the population of 13-24 year-olds in Nigeria and standard errors were adjusted for the complex sampling design. All analyses were conducted using Stata (version 14) [24].

## RESULTS

**Table 2** summarises key demographic characteristics and variables of interest across males and females. While males and females in the sample were approximately the same age, significant differences across sex were observed for all other variables. Compared to males, females were significantly more likely to be married (29% v. 9%; *P* < 0.0.01) and less likely to have ever attended school (78% v. 88%; *P* = 0.004). Male and female adolescents and young adults faced different exposures to violence victimisation as well. While past-year exposure to physical and emotional violence was greater among males (30.1% and 24.8%, respectively), females faced higher risks of experiencing sexual violence and IPV (15.5% and 8.0%, respectively). However, the data show that males were nearly twice as likely as females to have ever perpetrated IPV (9% v. 5%; *P* < 0.001). Finally, self-reported feelings of severe sadness and worthlessness in the last 30 days and ever considering suicide were more common among females (8.4% and 6.3%, respectively), and alcohol use was more likely to be reported by males (17.3%).

**Table 2:** Key characteristics, by gender

	Males	Females	Difference (*P*-value)
Age (mean in years)	18.2	18.5	0.080
Married	9.3%	29.0%	<0.001§
Ever attended school	87.7%	78.1%	0.004‡
Violence victimisation, last 12 months:			
Physical violence	30.1%	24.8%	0.016 †
Emotional violence	7.3%	4.6%	0.008‡
Sexual violence	10.0%	15.5%	0.001‡
IPV	3.5%	8.0%	<0.001§
Ever perpetration of IPV	8.6%	4.9%	<0.001§
Severe sadness, last 30 days	5.4%	8.4%	0.007‡
Worthlessness symptoms, last 30 days	1.9%	5.9%	<0.001§
Suicide ideation, ever	3.1%	6.3%	0.001‡
Number of days drunk, last 30 days	0.58	0.19	<0.001§

IPV – intimate partner violence

*Based on questionnaire construction, sexual intimate partner violence (IPV) is counted in both sexual violence and IPV. Standard errors are adjusted for complex sampling design. All observations are weighted to be representative of the population. Statistical significance of differences between groups are assessed using adjusted Wald tests.

†Difference significant at *P* < 0.05.

‡Difference significant at *P* < 0.01.

§Difference significant at *P* < 0.001.

When further examining the relationship between perpetration of IPV and feelings of severe sadness in the last 30 days, we found that females who reported having ever perpetrated IPV had 2.73 times higher odds of exhibiting severe sadness than females who did not report having ever perpetrated IPV (95% confidence interval (CI) = 1.44, 5.17; *P* = 0.002), even when controlling for exposure to violence victimisation in the last 12 months (see **Table 3**, Panel A). In contrast, no association between perpetration and severe sadness existed for males. Similarly, as shown in Panel C of **Table 3**, female perpetrators of IPV had 2.72 times greater odds of reporting ever having had suicide ideation as compared to females who had never perpetrated IPV (95% CI = 1.28, 5.79; *P* = 0.010), but this association was not significant for males.

**Table 3:** Perpetration of intimate partner violence (IPV), mental health symptoms, and alcohol use in Nigeria, adjusted odds ratios*

	Male		Female	
	**Model 1 (95% CI)**	**Model 2 (95% CI)**	**Model 1 [95% CI)**	**Model 2 [95% CI)**
**Panel A: Severe sadness:**	aOR	aOR	aOR	aOR
Perpetration of IPV, ever	1.77 (0.96, 3.28)	1.4 (0.76, 2.57)	2.60‡ (1.39, 4.87)	2.73‡ (1.44, 5.17)
Physical violence victimisation, past 12 months		1.60† (1.02, 2.49)		0.53† (0.31, 0.91)
Emotional violence victimisation, past 12 months		1.58 (0.82, 3.05)		1.1 (0.56, 2.15)
Sexual violence victimisation, past 12 months		1.93‡ (1.17, 3.16)		0.59 (0.33, .06)
IPV victimisation, past 12 months		0.51 (0.21, 1.25)		3.35§ (1.78,6.32)
**Panel B: Worthlessness symptoms:**	**aOR**	**aOR**	**aOR**	**aOR**
Perpetration of IPV, ever	2.73† (1.07, 6.95)	2.65† (1.09, 6.43)	1.21 (0.50, 2.92)	1.28 (0.52, 3.15)
Physical violence victimisation, past 12 months		1.58 (0.83, 3.04)		0.51 (0.25, 1.04)
Emotional violence victimisation, past 12 months		0.18 (0.02, 1.50)		1.25 (0.50,3.13)
Sexual violence victimisation, past 12 months		1.09 (0.36, 3.29)		0.38† (0.17, 0.83)
IPV victimisation, past 12 months		0.72 (0.13, 3.97)		3.65†† (1.64, 8.13)
Panel C: Suicide ideation, ever	aOR	aOR	aOR	aOR
Perpetration of IPV, ever	1.45 (0.54, 3.91)	0.90 (0.37, 2.19)	3.13‡ (1.50, 6.55)	2.72‡ (1.28, 5.79)
Physical violence victimisation, past 12 months		1.63 (0.87, 3.06)		1.80† (1.07, 3.05)
Emotional violence victimisation, past 12 months		1.98 (0.90, 4.37)		0.46 (0.15, 1.35)
Sexual violence victimisation, past 12 months		1.65 (0.69, 3.98)		2.70‡ (1.49, 4.87)
IPV victimisation, past 12 months		2.58 (0.82, 8.16)		1.75 (0.80,3.79)
Panel D: Drunk in last 30 days	IRR	IRR	IRR	IRR
Perpetration of IPV, ever	1.58† (1.01, 2.45)	1.63† (1.03, 2.57)	1.84 (0.81, 4.19)	1.71 (0.72, 4.05)
Physical violence victimisation, past 12 months		0.81 (0.50, 1.32)		1.76 (0.84,3.68)
Emotional violence victimisation, past 12 months		0.57† (0.34, 0.95)		0.68 0.36, 1.30)
Sexual violence victimisation, past 12 months		1.2 (0.72, 2.01)		0.96 (0.50, 1.87)
IPV victimisation, past 12 months		0.9 (0.36, 2.23)		1.66 (0.88, 3.13)

CI – confidence interval, aOR – adjusted odds ratio, IPV – intimate partner violence, IRR – incidence rate ratio

*Logistic regressions are used to estimate mental health symptoms and Poisson regressions are used to estimate number of days drunk in the last 30 d. Confidence Intervals (CI) are presented at 95%. Odds ratios are presented for panels A-C; incidence rate ratios (IRR) are presented for Panel D. All models control for age, marital status, and having ever been to school. Married females had lower odds of ever suicide ideation as compared to unmarried females (aOR = 0.30; *P* < 0.001). Age was significantly associated with alcohol use for males (aOR = 1.07; *P* = 0.001). Standard errors are adjusted for stratification and clustering.

†ORs and IRRs are statistically significant at *P* < 0.05.

‡ORs and IRRs are statistically significant at *P* < 0.01.

§ORs and IRRs are statistically significant at *P* < 0.001.

We observed a different pattern when examining associations between perpetration of IPV and symptoms of worthlessness and alcohol use (see **Table 3**, Panels B and D, respectively). While males who had ever perpetrated IPV had 2.65 times greater odds of reporting feelings of worthlessness as compared to males who had never perpetrated IPV (95% CI = 1.09, 6.43; *P* = 0.031), perpetration of IPV was not associated with this mental health symptom for females. Similarly, perpetration of IPV was associated with an increased rate ratio of alcohol use by a factor of 1.63 for males (95% CI = 1.03, 2.57; *P* = 0.037), but no such association was found for females.

Finally, all findings are robust to our sensitivity analyses, in which we estimate the same models subsampled on those individuals who have never experienced IPV.

## DISCUSSION

Given the significant mental health burden in Nigeria (and in LMICs more broadly), this study aimed to expand upon the available literature around adolescent and young adult violence and mental health by exploring the association of IPV perpetration and four mental health outcomes among male and female adolescents and young adults. Our findings add to the literature by demonstrating not only that violence perpetration is associated with a mental health burden but that this burden is gendered. Female perpetrators had increased odds of reporting suicide ideation and feelings of sadness, while male perpetrators had increased odds of reporting alcohol use and feelings of worthlessness. Further, our results hold even when controlling for IPV victimisation, a unique contribution of this paper.

By focusing on adolescents and young adults, our findings address a life stage often overlooked by research related to violence, and a period that can shape lifelong traits and behaviours [25]. When using a life cycle approach to examine risk factors for mental disorders, adolescence and young adulthood are often identified as formative periods during which exposure to violence, abuse, and neglect can influence the adult-onset of mental health problems [26,27]. Evidence also suggests that mental health issues that develop during adolescence and young adulthood can often persist later in life [28,29]. Further, adolescence is also a time when gender norms and attitudes are crystallizing, which may have implications for research and interventions looking to address violence and mental health [30]. There is emerging attention to the serious mental health needs of African adolescents and young adults, many of whom face daily challenges, including extreme poverty, conflict, displacement, illness, physical and sexual victimisation, or bereavement due to loss of parents to AIDS or non-communicable diseases, all of which can contribute to poor mental health [31-33]. A recent systematic review estimated that one in seven young people in sub-Saharan Africa may struggle with a mental health issue [31], and the World Health Organization estimates prevalence rates of ever experiencing a mental disorder during childhood or adolescence may be even higher (20%) [32,33]. Mental health policy is at an early stage, and specifically focused on the development of guidelines or identifying workforce shortages. Considered alongside the findings presented in this study, IPV perpetration in adolescence and young adulthood may have significant and lasting implications for perpetrators’ mental health well into adulthood. In addition to programming and policy linked to supporting the mental health needs of survivors of IPV, considering how best to respond to the different mental health needs of male and female perpetrators of IPV may lead to innovations in breaking cycles of violence in Nigeria and in other parts of sub-Saharan Africa.

Our findings align with the limited existing literature that suggests IPV has a mental health burden for perpetrators. Our results are also consistent with mental health literature that demonstrates gendered expressions of mental health. Two of our outcomes, worthlessness and severe sadness, are depressive symptoms, yet these were observed to differ in males, who expressed the former manifestation, and females, who reported the latter outcome. Our other outcomes are consistent with findings that females are more likely to report inward expressions of adverse mental health such as suicide ideation, while males are more likely to exhibit outward expressions of mental distress such as antisocial behaviour or substance use [34,35].

To aid in interpretation of these findings and discussion of their implications, we theorise about the potential influence of gender norms on these pathways. For males, traditional and ‘toxic’ views of masculinity may not only support perpetration of IPV but also dictate the ways in which male perpetrators express the toxic results of their actions. We note that feelings of low self-worth and alcohol use are also risk factors for violence victimisation and perpetration [36], which may suggest a negative feedback loop that results in further violence. Less researched are pathways and explanatory models that might help elucidate the links between female perpetration of IPV and resulting depressive symptoms and suicide ideation present in our sample. We hypothesise that in a patriarchal society such as Nigeria, when females transgress norms related to their expected roles as obedient and docile wives, there may be sanctions for perpetrating IPV [37,38]. It may that be the act of deviating from a strong gender norm and/or the associated repercussions for females are contributing to their mental distress.

Building on findings from this study, programming and policy would be wise to integrate gendered aspects of violence perpetration for both IPV prevention and response. While a growing number of IPV prevention programmes have started working with males and focusing on men’s exploration of “healthy masculinity”, they may fail to address the complex associations between triggers of violence and male-specific mental health issues [39,40]. Similarly, our findings for females suggest that policies and programmes targeting adolescents and young adults should thoughtfully consider whether and how women and girls may unintentionally suffer when acting counter to well-established inequitable gender norms in their communities. While challenging gender norms is generally accepted to be a positive endeavour (one could argue under what circumstances IPV perpetration by a female might be considered positive or negative), it is also recognized that early adopters of such behaviour may face consequences for challenging these norms [41].

Conclusions drawn from these findings should be considered alongside a few study limitations. First, all mental health outcomes assessed in this study are derived from single-question, self-reported responses as opposed to mental health scales. Second, given the cross-sectional nature of the data, the lack of temporality makes it difficult to assert causality with complete confidence. This limitation is particularly relevant for the suicidal ideation outcome, which is defined as having *ever* considered suicide, due to available questions in the VACS. Finally, given restrictions in the data, this analysis does not disaggregate data across the six geopolitical zones. As such, interpretation of the findings cannot shed light on how these associations may be especially pronounced or muted in certain geographical areas. Nonetheless, our study utilised a robust, nationally representative data set and represents the first of its kind to explore the gendered associations between IPV perpetration and mental health for adolescents and young adults in an LMIC.

## CONCLUSIONS

The findings from this research highlight a relatively new topic that, if further validated across contexts, would have important implications for policy and programming. Considering the compelling and gendered findings from this study, we urge the research community to further explore causal pathways between IPV perpetration and mental health outcomes. Finally, we advocate for the integration of gendered considerations within IPV programming and policies for adolescents and young adults. Responses, especially those tailored to female adolescents and young adults, should be mindful of how gender norms may influence the association of IPV perpetration and mental health outcomes, as well as the direction of these pathways.
